# Transition of patients with recently diagnosed Dementia from inpatient to outpatient setting– a scoping review

**DOI:** 10.1186/s12877-023-04638-y

**Published:** 2024-01-08

**Authors:** Flora-Marie Hegerath, Chantal Giehl, Michael Pentzek, Horst Christian Vollmar, Ina Carola Otte

**Affiliations:** 1 Institute of General Practice and Family Medicine (AM RUB), Medical Faculty, Ruhr University Bochum, 44801 Bochum, Germany; 2 Institute of Family Practice, Medical Faculty, University of Duisburg-Essen, 45147 Essen, Germany

**Keywords:** Scoping review, Dementia, Alzheimer’s disease, Primary care, Hospital, Discharge management, Communication, Health services research, Germany

## Abstract

**Introduction:**

After being diagnosed with dementia, patients need a medical professional to empathetically address their fears and get initial questions answered. This scoping review therefore addresses how patients newly diagnosed with dementia are cared for in the general practitioner (GP) setting and how the communication between different healthcare professionals and the GP is handled.

**Methods:**

The scoping review was conducted based on the PRISMA Extension for Scoping Reviews checklist. After developing a search algorithm, literature searches were performed in PubMed, Scopus, Web of Science, Cochrane Library, PsychInfo, GeroLit and Cinahl using defined search criteria, such as a focus on qualitative study designs. After the removal of duplicates, title/abstract and full text screening was carried out.

**Results:**

Final data extraction included 10 articles out of 12,633 records. Strategies regarding the post-acute care of newly diagnosed patients included providing clarity and comfort to the patients and giving support and information both pre- and post-diagnosis. Care efforts were focused on advanced care planning and deprescribing. Involving people with dementia and their caregivers in further care was seen as crucial to provide them with the support needed. GPs emphasised the importance of listening to concerns, as well as ensuring wishes are respected, and autonomy is maintained. All studies found communication between the GP setting and other healthcare professionals regarding post-acute care to be inadequate. Lack of information sharing, clinical notes and recommendations for the GP setting resulted in inefficient provision of support, as GPs feel limited in their ability to act.

**Discussion:**

Sharing necessary information with the GP setting could promote patient-centred care for people living with dementia and facilitate appropriate and timely resource allocation and effective healthcare collaboration between the settings, for example, by defining clear care pathways and clarifying roles and expectations.

**Supplementary Information:**

The online version contains supplementary material available at 10.1186/s12877-023-04638-y.

## Introduction

Facing a diagnosis of dementia, patients and their families are confronted with various challenges, such as the possible decline and loss of cognitive function and daily living skills, the prognosis of extensive care needs in the future [1], and in general insufficient provision of medical information by healthcare professionals (HCPs) [2]. Those affected emphasise the importance of receiving post-diagnostic support quickly. This support needs to be effective and tailored to their individual needs and circumstances [3].

People with dementia (PwD) and their caregivers further wish for a single HCP to act as a reference throughout the entire course of the disease [4]. In Germany, general practitioners (GPs) usually carry out this function and act as a navigator in the healthcare system for planning further care. GPs are often the first point of contact for health issues and therefore play an important role as confidants, especially for older people, based on a long-term, well-established patient-doctor relationship [5, 6]. GPs are expected to take a central role and act as case managers in the diagnosis process and subsequent care of PwD [7]. A post-diagnostic discussion about dementia in the GP setting should therefore clarify fears and questions, verify the diagnosis and initiate appropriate actions [8, 9].

To fulfil their role and support patients efficiently, GPs rely on necessary information from other HCPs involved in diagnosis and care. As communication between healthcare settings is seen as insufficient especially when patients with an acute health issue are transitioned from inpatient to outpatient setting [4] the aim of the review was to identify relevant articles that focus on the post-diagnostic care of PwD in general practice. We therefore focused on patients who have received their diagnosis outside of the GP setting, e.g. during an acute hospital stay [10]. The research team developed the following research questions:

1) How are patients newly diagnosed with dementia cared for in general practice?

2) How is the communication between the different settings and the GP practice handled?

## Materials and methods

This scoping review was carried out in accordance with the PRISMA Extension for Scoping Reviews checklist [11]. In addition, the review is guided by the enhanced recommendations of the methodological framework by Arksey and O’Malley implemented by Levac, Colquhoun and O’Brien [12, 13]. Therefore the authors followed the five-stage-approach including: identifying the research question, identifying relevant studies, study selection, charting the data and collating, summarising and reporting the results [12]. According to Arksey and O’Malley, research questions are supposed to maintain a broad scope and combine a broad research question with a clearly formulated scope of inquiry [12, 13]. On this basis, the research team developed the research questions mentioned in the introduction.

This review is part of the qualitative study “MeDeKa - Primary care for people newly diagnosed with dementia after hospital discharge” (DRKS-ID: DRKS00025061) which is funded by the German Alzheimer Society. The protocol of the review is registered at Open Science Framework [10].

### Identifying relevant studies

The comprehensive literature search was performed by the two main researchers (CG, FMH) in the following databases: PubMed, Scopus, Web of Science, Cochrane Library, PsychInfo, GeroLit and Cinahl. The main researchers developed search terms based on common literature in this area [14–16]. Search terms regarding qualitative studies were developed based on topic-specific PubMed queries for Health Services Research (MeSH Unique ID: D036301). The research team (CG, FMH, HCV, IO) discussed the final search terms, expanded and adapted them for each database.

The publication type was focused on journal articles with a qualitative study design or reviews to provide a comprehensive overview of the perceived barriers and facilitators caring for people newly diagnosed with dementia. Qualitative studies help to interpret contexts of meaning, taking into account the respective setting [17, 18].

We included articles in English and German. In addition, we made no restriction on the year of publication. The adjusted search algorithms for each database can be found in the additional file 1.

The research team carried out the literature searches at two points in time: The first search in May 2021, the second in November 2021, to include new articles that may have been published since May 2021.

### Study selection

For the title and abstract screening, the main researchers divided the seven databases between them. To not exclude potentially important studies, exclusion only took place in case of clear deviation from the study aim and if quantitative study methods were used. When in doubt, the researchers included articles for full text screening. The inclusion and exclusion criteria are given in Table 1.

In the second search in November 2021, the main researchers used the same search algorithms and databases. Each researcher carried out the search in the databases in which they conducted the initial search. Newly published articles were added to the existing dataset and duplicates were removed. The screening of title and abstracts was carried out seperately by the main researchers. The full text screening was carried out jointly by the main researchers. Disagreements and potential conflicts were discussed within the research team. In a next step, an additional hand search was conducted examining the literature cited in the studies included.

**Table 1:** Inclusion and exclusion criteria

Inclusion criteria	Exclusion criteria
-transition of patients with recently diagnosed dementia from inpatient to outpatient setting -diagnosed dementia -management of newly diagnosed dementia/delirium -general practice -involvement of family caregivers in the process of caring -qualitative study design	-focus on diseases other than dementia or delirium -primary focus on diagnostic tool -primary focus on health of caring relatives -quantitative study design

### Charting the data

A form adapted to PRISMA-ScR scheme was used for data extraction. Extracted information included: authors, country, aim, study design and type of study, setting, target population, participants’ characteristics, measures and outcomes, diagnosis, key results, suggestions in caring for PwD, potential barriers caring for patients (GPs view), involvement of PwD and their caregivers in further care, funding, study limitations, ethics approval and concerns and others. The main researchers independently extracted the data and proofread each other’s extracted data.

### Collating, summarising, and reporting results

Due to the broad scope of the review, a wide range of findings regarding the research questions were included and summarised in tabular and narrative form. The main researchers met a total of 20 times within five months for one hour each time to discuss the extracted data in context of the aim of the review. Columns were added or merged to enable a more concise presentation and to analyse the data appropriately.

## Results

**Fig. 1:**
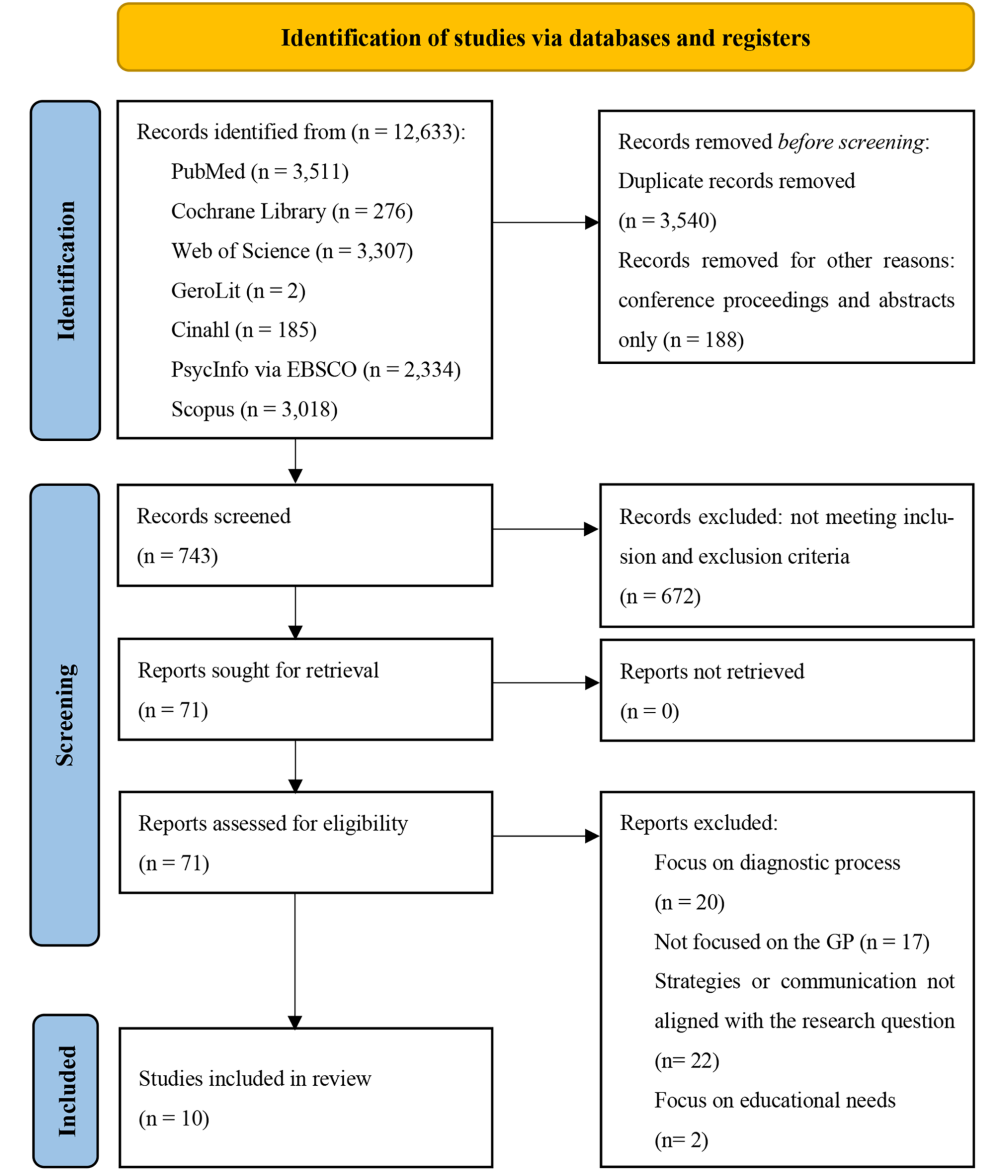
PRISMA flow diagram [20]

Results of the search strategy are presented in the PRISMA flowchart (Fig. 1). Through database searches, the main researchers identified a total of 12,633 records within the two searches. Of these, 71 full texts met the inclusion criteria in Table 1.The final data extraction included 10 articles.

### Characteristics of included studies

Three studies were conducted in England (with one of the three studies in England and Wales). Two studies were carried out in Australia. One study each was conducted in Ireland, USA, Canada, Spain, and UK. All studies were published in English. As one inclusion criteria was a qualitative study design, most studies had a qualitative study design (n = 9). One study was a systematic review of qualitative studies [19].

Settings of the studies included varied between different healthcare sectors, e.g. GP setting, hospital, nursing homes and social care. One qualitative study did not mention the setting and in the review of qualitative studies a definition of the setting was not applicable. The target population varied between the different studies: GPs, HCPs, PwD and their caregivers were included. Detailed information regarding the population is given in Table 2.

As the aim of this review was examine the transition of newly diagnosed patients from one healthcare sector to another, extracted data included place and time of diagnosis. Only three studies reported information on this in varying degrees of detail. Detailed information regarding the diagnosis is given in Table 2.

Within the studies, the generalisability of the findings was highlighted as a limitation due to the qualitative study design [4, 7, 19, 21–26]. Eight studies disclosed their funding. Two studies did not mention funding. All but one study obtained ethical approval.

**Table 2:** Studies included in the scoping review

Authors/ country	Design	Setting	Target population	Participants	Measures and Outcomes	Diagnosis	Strategies and approaches in GP setting	Communication between HCPs
Bourque, M., et al. (2020), Ireland	Qualitative	Department of General Practice	GPs	n = 12, female (n = 7), age of majority (n = 10) 40–59 years, average experience 17.2 years, mixed urban-rural settings (n = 9)	Interviews to understand how to improve the quality of dementia care		Following a diagnosis, GPs reported their role was ad hoc and reactive. GPs care efforts focused on advanced care planning and deprescribing	GPs collaborate with care providers from different settings, mentioning lack of coordination among community services
Burn, A., et al. (2019), England	Qualitative	Acute hospital	PwD aged ≥ 75 years who had been hospitalised 6–12 months previously and had been diagnosed with dementia	n = 49, patients (n = 24), female (53%), median and mean age 85 (range 79–94), carers (n = 25)	Interviews to gather patients’ and caregivers’ experiences of dementia case finding in hospital	Unclear whether all participants received a diagnosis; possibly during hospitalisation or after discharge	Case finding did not necessarily lead to GP follow-up	
Burn, A., et al. (2018), England	Qualitative	Hospital and GP setting	Hospital staff involved in dementia case finding and primary care staff in the catchment areas of those hospitals	n = 59, primary care stuff (n = 36) including GPs (n = 30), average clinical experience 22.5 years of participants in focus group	Interviews and focus group discussions to explore views on benefits and challenges of case finding in hospitals	In Hospital		Poor communication of case finding information from secondary care to primary care. GPs do not receive much information from the hospital. The ability to act is therefore limited
Hinton, L., et al. (2007), USA	Qualitative	GP setting	GPs	n = 40, male (n = 35), age of majority (n = 23) 46–65 years, white non-hispanic (n = 27)	Interviews to explore view on practice constraints and how they affect appropriate care		Some GPs wanted psychiatrists’ opinions on managing behavioural problems. Others preferred ongoing care by a specialist. Family members are often consulted as a primary source for history taking, to assist in decision making and development of treatment plans	Communication is often difficult due to limited availability of specialists and lack of “feedback” in the form of clinical notes and recommendations for the GP. This would allow GPs to discuss the recommendations with the patient and family
Hum, S., et al. (2014), Canada	Qualitative	Academic health sciences centres, community academic hospitals	GPs and specialists	n = 12, GPs (n = 6), female (n = 4), average experience 17.5 years, specialists (n = 6)	Interviews to explore the perceived roles and attitudes towards dementia care from the perspectives of GPs and specialists		GPs show greater confidence in initial management of dementia and refer to a specialist for management of behavioural and psychiatric symptoms of dementia, for prescribing psychotropics, for diagnosis of atypical dementias, management of complex, co-morbid conditions, or a patient or a family caregiver’s request	Communication is usually one-way. Interactions between GPs and specialists depend on the individual physicians
Risco, E., et al. (2016), Spain	Qualitative		PwD, HCPs caregiver	n = 37, PwD (n = 7), female (n = 4), mean age 74.2 years; family caregivers (n = 11), mean age 78.3 years, wives (n = 7), husbands (n = 3), son (n = 1); HCPs (n = 19) including GPs (n = 4), mean age 41.2 years	Focus group discussions to identify barriers and facilitators in dementia care			Inadequate communication between HCP, especially when PwD move from one provider to another
Robinson, A., et al. (2009), Australia	Qualitative	GP setting, nursing service, nursing homes, community	GPs, HCPs	n = 84, GPs n = 7, other HCPs (n = 77)	Focus group discussions to address issues related to availability and transfer of information and information needs			Lack of communication between service providers leading to inefficiencies in service provision. Most providers complain about an uncoordinated service delivery system due to the lack of information sharing
Tuijt, R., et al. (2021), UK	Systematic review of qualitative studies		PwD, HCPs caregiver	29 included papers concerning 27 studies. PwD n = 261 (median 7 per study), carers n = 444 (median 11.5), HCPs including GPs n = 530 (median 12)	Identify the experiences of health care services as well as facilitating or hindering factors for functioning triads		Inclusion of PwD is beneficial, especially in early stages when planning is needed. Adapt activities to maintain independence of PwD and “acceptable” risks were tolerated if they were thought to improve mental and physical well-being. Establishing a triad in dementia care	Good communication among professionals improved support and treatment of PwD, as well as facilitated appropriate and timely resource allocation and effective collaboration and care coordination
Walker, R., et al. (2018), Australia	Qualitative	Home setting	PwD with mild dementia, caregiver	n = 16, PwD (n = 9), male (n = 5), average age 80 years caregivers (n = 7)	Interviews to determine how PwD and caregiver experience dementia assessment services	Diagnosis within the last three months; not made in primary care	GP as an important contact during assessment of dementia providing clarity and comfort, giving support and information both pre- and post-diagnosis	
Wheatley, A., et al. (2021), England and Wales	Qualitative	primary care, secondary mental health, third sector, social care, NHS clinical commissioning groups	GPs, HCPs, PwD, caregiver		Interviews, focus group discussions, observation to explore barriers to providing post-diagnostic support			Effective communication and coordination among health care professionals are crucial to provide high-quality post-diagnostic support

### Strategies used in GP setting

Six studies mentioned strategies in the GP setting regarding post-acute care of newly diagnosed patients who were not diagnosed in the GP setting. In the study of Bourque et al. (2020), GPs reported that their role following a formal diagnosis of dementia was “initially ad hoc and reactive”. Care efforts focused on advanced care planning and deprescribing [27]. GPs see themselves as an important contact in the assessment of dementia. Strategies mentioned were providing clarity and comfort to the patients and offering support and information pre- and post-diagnosis. Family members and other informants were often helpful as a primary source of history and to assist in decision making and planning for future care [25]. Leading up to the diagnosis GPs referred the majority of their patient to a specialist geriatrician [26]. GPs further refer patients to a specialist for management of behavioural and psychiatric symptoms of dementia [7, 25].

Strategies mentioned in the study of Wheatley et al. (2021) mainly focus on service managers and commissioners but are including GPs. Involving PwD and their caregivers in further care is crucial to provide them with the support they need. Particiants in this study emphasised the importance of listening to concerns and being responsive to needs of PwD and caregivers, as well as ensuring that their wishes are respected and that their autonomy is maintained [21].

In the review of Tuijt et al. (2021) the establishment of a triad in dementia care was emphasised. Involving PwD in this triad was seen as beneficial, especially in the early stages of the disease when planning further care. To improve independence, activities were modified to keep them accessible to the PwD. Thereby acceptable risks were tolerated if they were thought to improve mental and physical wellbeing. Another strategy mentioned in the review was enabling socialisation of the PwD, as it counteracted loneliness, helped individuals find support, maintain a sense of identity and give them a sense of achievement [19].

### Communication between different HCPs and the GP practice

Eight studies provided heterogeneous information on communication between GPs and different HCPs regarding the post-acute care of newly diagnosed patients. In general communication was seen as insufficient throughout the studies.

The study of Hinton et al. (2007) emphasised the difficult communication between different HCPs due to a limited availability of specialists. GPs mentioned a lack of clinical notes and recommendations for the GP setting from other HCPs. This information would allow GPs to discuss the specialist’s recommendations with the patient and family [25].

Burn et al. (2018) mentioned that GPs did not receive much information from the hospital, when case-finding took place. Discharge reports were inconsistent and did not include essential information, such as the type of cognitive assessment used, the patient’s assessment score or instructions for further care and referral. For this reason GPs were limited in their ability to act [24].

Participating PwD, caregivers and HCPs in the study of Risco et al. (2016) mentioned a generally insufficient level of communication between HCPs. This was common, when PwD changed from one provider to another especially when acute problems appear and the person being cared for at home needs to be admitted to a hospital [4]. Communication was generally one-way and the interactions between GPs and specialists depended on the individual physician [7].

In focus group discussions in the study of Robinson et al. (2009) HCPs and GPs mentioned a lack of communication and sufficient information between providers that led to inefficiencies in service delivery. HCPs need to gather and piece together information, e.g. with the help of family members, however, this can lead to misinformation for a variety of reasons [22]. Bourque et al. (2020) also highlighted a lack of coordination among community services [27]. Effective communication and coordination between HCPs was highlighted as crucial to provide high quality post-diagnostic support [21].

The review of Tuijt et al. (2021) emphasised that providers communicating well with each other improved the support and treatment for the PwD and seemed to facilitate appropriate and timely resource allocation as well as effective collaboration and the coordination of care [19].

### Potential barriers caring for patients (GPs view)

Eigth articles mentioned further barriers in caring for PwD. GPs highlighted challenges in dementia care due to lack of time [7, 21, 27] and funding to provide structured care [7, 25, 27]. A mismatch between the number of consultations and the workload involved in dementia care in General Practice was mentioned [27] as caring for PwD is more time intensive for several additional reasons [25]. As the patient’s clinical condition deteriorates, they require more frequent and intensive care [27]. Another obstacle to care arises when PwD do not accept their diagnosis. This disrupts the work of professionals and the initiation and establishment of a triad [19].

Other barriers occurred within the structure of the health care system. In general, limited structural resources in dementia care were mentioned [19]. Further barriers mentioned were fragmented and difficult to access community resources as well as medical management of behavioural and psychiatric symptoms of dementia [7]. Infrastructure of secondary care services was considered insufficient due to lack of coordination between community services [21, 27]. Initiating person-centred care was seen as challenging [25] due to a lack of, for example, clear care pathways and access to specialist support [21]. The use of different assessment tools in secondary and primary care complicates the interpretation of test scores [24].

The study by Burn et al. (2019) focused on experiences of dementia case finding in hospital from the perspective of PwD and caregivers. As mentioned from participants, case finding did not necessarily lead to GP follow-up after discharge or to referral for further examinations post discharge [23]. Hospitals were considered an inappropriate setting for case finding because acute illness, medications, or delirium could lead to low or inaccurate assessment scores [24]. A problematic factor in clear diagnosis was when suspicion of dementia arose during hospitalisation for another condition (e.g. a fall) [26].

GPs indicated inadequate training in dementia care [19] and a lack of knowledge to assist families in accessing social services. A sense of frustration may occur when GPs feel compelled to provide care that they feel is outside their area of expertise [25].

### Suggestions in caring for PwD

Nine studies mentioned suggestions in caring for PwD. To optimise communication within the health care system and address the mentioned barriers, authors mentioned the establishment of shared care pathways [21]. Written agreements specifying the responsibilities of different HCPs in relation to patient care could be one way to clarify roles and expectations. An agreement on acute support for complex problems by a specialist could support care in the GP setting [21]. Information from hospitals to primary care should be comprehensive, appropriate for the setting and consistent in order to effectively plan further care [24].

Because of the social stigma associated with dementia, discussions about the diagnosis should be conducted by trained and qualified staff [24]. Changes in communication strategies between the professional and the PwD such as asking shorter questions or taking more time could improve the involvement of PwD [19].

Other recommendations focus on the establishment of a new practice framework for the optimal provision of relevant information for PwD and caregivers depending on the stage of illness in the context of person-centred care [4]. A standardised assessment scheme could help primary care physicians expedite the diagnostic process [7]. More obvious cases of Alzheimer’s disease could then be diagnosed by GPs themselves [7].

Dementia care can be improved by introducing a structured care programme in primary care, improving community resources, formalising local dementia networks and standardising dementia resources [27]. A collaboration between physicians and non-profit organisations, such as the Alzheimer’s Society, could enhance coordinated provision of information on resources and service providers to minimise confusion about fragmented resources and access to community and social support services [7]. One option for improving collaboration in the health sector would be an electronic database of services. Better communication between providers could prevent valuable information from being lost [22]. An additional way could be the adoption of new communication platforms to support communication and collaboration by sharing medical records [4].

To meet the needs of PwD and caregivers, educational interventions for families and GPs and broader structural changes are necessary [25] as well as ongoing training for GPs [27].

## Discussion

The aim of this scoping review was to examine how patients newly diagnosed with dementia are cared for in the GP setting and how the communication between different healthcare settings and the GP setting is handled. There has been little research on how people newly diagnosed with dementia are cared for in general practice.

To discuss the results presented in this scoping review, we use a framework of dividing the healthcare system into the macro, meso and micro levels. By using this approach, we are able to assign our results to the corresponding levels, which in return clarifies in what form obstacles arise.

At the macro level, and therefore at the top of the healthcare system, the state´s institutions play a crucial role. On this level, the framework and legal conditions of the healthcare system are defined. At the meso level, we find organisations and institutions that further specify the legal requirements of the state. The statutory regulations are concretised, for example, within guidelines and collective agreements. As a result, the organisations and institutions coordinate the providers in the healthcare system and define the care objectives. At the micro level, care recipients and care providers interact with each other as well as care providers interact with one another [28].

In the scoping review, several studies criticised the respective structure within the healthcare systems, due to limited structural resources in dementia care [19], fragmented and difficult to access community resources, a lack of clear care pathways and access to specialist support [7, 21] and a lack of coordination between community services resulting in an insufficient infrastructure of secondary care services [21, 27]. Overall, these results highlight the challenges at the different levels of the healthcare system. Structural deficits and limited resources at the macro level contribute to fragmented community resources at the meso level, resulting in challenges in patient care at the micro level. Based on these results of the scoping review, we gain insight into the close interaction between the levels in day-to-day care [28].

The scoping review by Martin et al. (2020) on gaps and priorities in dementia care in Europe also identified often poorly coordinated dementia care. The healthcare systems are fragmented and not designed for interactions between the different parts of the system which makes it difficult to navigate for PwD. Ineffective care pathways lead to unnecessary interventions and referrals, as well as poor experiences of care. Other countries and healthcare systems outside of Europe face similar problems, as this scoping review has highlighted [29].

Clear care pathways were mentioned as a promising way to provide sustainable care for PwD and could improve the communication within the healthcare system [21] and clarify roles and expectations [30]. It is an approach to the organisation and delivery of care that involves standardising and coordinating care across different providers and settings [30]. National dementia strategies around the world emphasise the importance of clear dementia pathways to support PwD and caregivers. It has been shown that care pathways can improve the quality of care and reduce costs, but attention must be paid to the challenges to their effective implementation [30]. Interprofessional community partnerships are seen as improving dementia care and meeting the diverse and complex needs of PwD and their caregivers [31]. Therefore collaboration between physicians and non-profit organisations on the micro level could enhance coordinated provision of information on resources and service providers [7]. Due to structural differences within healthcare systems, variations between the countries regarding the conditions under which care pathways are being implemented occur [30]. Consequently, transnational conclusions cannot be drawn.

Further, as each patient’s needs and circumstances are unique, no standardised pathway is suitable for all patients. It is therefore recommended that care pathways should be flexible and based on the needs and goals of the individual patient [30]. To improve collaboration and coordination in the care of PwD, interprofessional education for GPs and other HCPs is needed [32].

The results on the strategies and approaches of GPs caring for PwD are mainly based on the micro level. Strategies for caring for PwD were heterogeneous across the articles included in this scoping review. Following a diagnosis, GPs initially see their role as being “ad hoc and reactive” [27]. Care efforts were focused on advanced care planning and deprescribing [27], as well as providing clarity, support and information [26]. Involving PwD and their caregivers in planning further care and listening to concerns was seen as crucial in providing them with the support needed [19, 21, 25].

The results regarding the communication between GPs and HCPs can also be linked to the micro level. The results of the review highlight that communication between the GP setting and other HCPs regarding post-acute care was found to be inadequate throughout the studies. Lack of information sharing, clinical notes and recommendations for the GP setting resulted in an inefficient provision of support [22], as GPs felt limited in their ability to act [24, 25]. This again illustrates the way in which the state defines the legal framework at the macro level and how this framework is concretised by the organisations and institutions at the meso level, ultimately influencing the behaviour of the care recipients and care providers at the micro level. This influence works reciprocally, which means it does not necessarily needs to start top down but can also work bottom up - micro to the meso and from there to the macro level [28]. Proficient communication seemed to facilitate appropriate and timely resource allocation, as well as effective collaboration and the coordination of care [19]. Therefore information sharing between settings and coordination between HCPs would improve post-diagnostic support and in general the support and treatment for PwD [19, 21].

Sharing necessary information and medical records with the GP through a communication platform could promote exchange and collaboration [4]. For example, emergency data management could be used to bundle relevant patient data. With the patient’s consent, the data set could contain information on illnesses, surgery, medications, allergies and intolerances, important medical information as well as contact details of doctors and relatives [33]. A standardised electronic medication plan in the respective healthcare system can further increase drug therapy safety for patients. The plan contains personal information, application-specific information, intolerances, allergies, as well as the possibility of providing instructions for other HCPs involved in care [33].

Another option for improving collaboration in the healthcare sector could be an electronic database of services [22]. The Alzheimer Societies, for example, provide a catalogue of services for PwD. This could be expanded to include volunteer and medical services. Collaborating with HCPs could ensure that PwD are aware of these services.

## Limitations and strengths

This scoping review provides insight into the care of people who have received a dementia diagnosis. The focus on qualitative studies, while providing a deeper understanding of interrelationships and context, must also be seen as a limitation, as studies were potentially excluded. As a scoping review does not assess the quality of the evidence, the strategies used in GP setting cannot be comprehensively assessed for practice [34]. Furthermore, the research team decided against a targeted search for grey literature to ensure the reproducibility of the results. In addition, the quality of grey literature is not always comprehensible and thus limits the ability to replicate the study. Excluding grey literature can be seen as a limitation.

A possible limitation may be the broad scope of the topic, which led to the inclusion of studies with diverse settings and outcomes. Despite - or perhaps because of - the heterogeneity of the data, the review provides a good overview of the topic. Furthermore, due to structural differences in the healthcare systems of different countries no general statements can be made.

In conducting the scoping review the authors followed the five-stage-approach by Arksey and O’Malley, which is a feature of good scientific practice. During the review process, the authors followed the requirements of the framework and were thus able to ensure a good quality of the approach and the data presented. In addition, the topic of the review has a high practical relevance, as it highlights the problem of the transition of patients from the inpatient to the outpatient sector.

## Conclusion

GPs see themselves as an important contact in the assessment and care of PwD. They emphasised the importance of listening to their concerns and being responsive to their needs. Effective communication and collaboration within the healthcare system would be beneficial in this regard. Sharing necessary information with the GP setting, for example, by using compatible digital communication tools, could promote patient-centred care and facilitate appropriate and timely resource allocation and effective collaboration between healthcare settings.

The scoping review provides an overview of strategies used in the GP setting and provides insight into the communication between healthcare settings. However, the results presented only scratch the surface of the issue. Best practice can be used, for example, to improve the care of PwD in Germany. The extent to which this can be implemented is the subject of further research. Further research should delve deeper into the needs of GPs to provide optimal care.

### Electronic supplementary material

Below is the link to the electronic supplementary material.

Supplementary Material 1

Supplementary Material 2

## Data Availability

The datasets used and analysed during the current study are available from the corresponding author on reasonable request.

## References

[1] DGN e. V. & DGPPN e. V., Herausgeber S3-Leitlinie Demenzen, Version 4.0, 8.11.2023.Available from: https://register.awmf.org/de/leitlinien/detail/038-013, last access on 01.12.2023.

[2] Zubatsky M, Aragon-Prada M, Muse F, Rainey P, Martin R (2016). Navigating without a Roadmap: challenges of early Alzheimer’s caregivers with their Health Care Team. Glob Qual Nurs Res.

[3] Wheatley A, Bamford C, Brunskill G, Harrison Dening K, Allan L, Rait G (2020). Task-shifted approaches to postdiagnostic Dementia support: a qualitative study exploring professional views and experiences. BMJ Open.

[4] Risco E, Cabrera E, Farré M, Alvira C, Miguel S, Zabalegui A (2016). Perspectives about Health Care Provision in Dementia Care in Spain: a qualitative study using Focus-Group Methodology. Am J Alzheimers Dis Other Demen.

[5] Pentzek M, Michel JV, Ufert M, Vollmar HC, Wilm S, Leve V (2015). Fahrtauglichkeit Bei Demenz - Theoretische Rahmung Und Konzept Einer Vorgehensempfehlung für die Hausarztpraxis. Z Evid Fortbild Qual Gesundhwes.

[6] Wagner G, Abholz HH (2002). Diagnose und Therapiemanagement Der Demenz in Der Hausarztpraxis. Z für Allgemeinmedizin.

[7] Hum S, Cohen C, Persaud M, Lee J, Drummond N, Dalziel W (2014). Role expectations in Dementia care among family physicians and specialists. Can Geriatr J.

[8] Vollmar HC, Wilm S, Kuske S, Gallrach F, Buscher I, Abholz HH et al. Changing attitudes towards dementia in family practice (CADIF) - evidence-based development and pre-testing of an intervention for general practitioners. In: Thyrian JR, Hoffmann W, editors. Dementia Care Research. Scientific Evidence, Current Issues and Future Perspectives: Minutes from an International Workshop in Greifswald.; 2012. p. 80–9.

[9] Pentzek M, Vollmar HC, Wilm S, Leve V (2017). Frühwahrnehmung Von Demenzen in Der Hausarztpraxis: Der CADIF-Ansatz. Z Gerontol Geriatr.

[10] Hegerath F-M, Giehl C, Vollmar HC, Otte I. Following up on recently diagnosed Dementia patients– implications for general practice: a scoping review. A study protocol; 2022.

[11] Tricco AC, Lillie E, Zarin W, O’Brien KK, Colquhoun H, Levac D (2018). PRISMA Extension for scoping reviews (PRISMA-ScR): Checklist and Explanation. Ann Intern Med.

[12] Levac D, Colquhoun H, O’Brien KK (2010). Scoping studies: advancing the methodology. Implement Sci.

[13] Arksey H, O’Malley L (2005). Scoping studies: towards a methodological framework. Int J Soc Res Methodol.

[14] Fox B, Henwood T, Neville C, Keogh J, Hodgkinson B (2015). The psychometric viability of measures of functional performance commonly employed for older adults with Dementia: a systematic review of measurement properties protocol. JBI Database System Rev Implement Rep.

[15] Gill PJ, Roberts NW, Wang KY, Heneghan C (2014). Development of a search filter for identifying studies completed in primary care. Fam Pract.

[16] Pols DHJ, Bramer WM, Bindels PJE, van de Laar FA, Bohnen AM (2015). Development and Validation of Search filters to identify articles on Family Medicine in Online Medical databases. The Annals of Family Medicine.

[17] Fossey E, Harvey C, Mcdermott F, Davidson L (2002). Understanding and evaluating qualitative research. Aust N Z J Psychiatry.

[18] Marx G, Wollny A (2009). Qualitative sozialforschung– qualitative sozialforschung– ausgangspunkte und Ansätze für eine forschende Allgemeinmedizin: teil 1: Theorie Und Grundlagen Der Qualitativen Forschung. Z für Allgemeinmedizin.

[19] Tuijt R, Rees J, Frost R, Wilcock J, Manthorpe J, Rait G et al. Exploring how triads of people living with dementia, carers and health care professionals function in dementia health care: A systematic qualitative review and thematic synthesis. Dementia. 2021; 20(3):1080–104. Available from: URL: https://www.scopus.com/inward/record.uri?eid=2-s2.0-85082417573&doi=10.1177%2f1471301220915068&partnerID=40&md5=e460f6be99a4a602ac0eaaf398f95f4b. 10.1177/1471301220915068 PMC8047709 32212862

[20] Page MJ, McKenzie JE, Bossuyt PM, Boutron I, Hoffmann TC, Mulrow CD (2021). The PRISMA 2020 statement: an updated guideline for reporting systematic reviews. BMJ.

[21] Wheatley A, Bamford C, Brunskill G, Booi L, Dening KH, Robinson L (2021). Implementing post-diagnostic support for people living with Dementia in England: a qualitative study of barriers and strategies used to address these in practice. Age Ageing.

[22] Robinson A, Emden C, Lea E, Elder J, Turner P, Vickers J (2009). Information issues for providers of services to people with Dementia living in the community in Australia: breaking the cycle of frustration. Health Soc Care Community.

[23] Burn A-M, Bunn F, Fleming J, Turner D, Fox C, Malyon A (2019). Case finding for Dementia during acute hospital admissions: a mixed-methods study exploring the impacts on patient care after discharge and costs for the English National Health Service. BMJ Open.

[24] Burn A-M, Fleming J, Brayne C, Fox C, Bunn F (2018). Dementia case-finding in hospitals: a qualitative study exploring the views of healthcare professionals in English primary care and secondary care. BMJ Open.

[25] Hinton L, Franz CE, Reddy G, Flores Y, Kravitz RL, Barker JC (2007). Practice constraints, behavioral problems, and Dementia care: primary care physicians’ perspectives. J Gen Intern Med.

[26] Walker R, Ratcliffe J, White A, Visvanathan R (2018). Dementia assessment services: what are the perceptions of older people?. Australas J Ageing.

[27] Bourque M, Foley T (2020). Improving the quality of Dementia Care in General Practice: a qualitative study. Front Med (Lausanne).

[28] Wessels M (2019). Pflegeökonomie.

[29] Martin A, O’Connor S, Jackson C (2020). A scoping review of gaps and priorities in Dementia care in Europe. Dement (London).

[30] Samsi K, Manthorpe J (2014). Care pathways for Dementia: current perspectives. Clin Interv Aging.

[31] Lee L, Hillier LM, Gregg S (2019). Partnerships for improving Dementia care in primary care: extending access to primary care-based memory clinics in Ontario, Canada. Health Soc Care Community.

[32] Dreier-Wolfgramm A, Michalowsky B, Austrom MG, van der Marck MA, Iliffe S, Alder C (2017). Versorgungsmanagement Bei Demenz in Der Primärversorgung: Aktuelle Kooperative Versorgungsmodelle Und Der Vorteil Von Interprofessionellem Lernen. Z Gerontol Geriatr.

[33] Steinhäuser J, Detmer J, Eichelberg M, Feldmeier G, Flägel K, Freiberg P, et al. editors. Telemedizin und eHealth: Das Wichtigste für Ärztinnen und Ärzte aller Fachrichtungen. 1. Auflage. München: Elsevier; 2021. (Elsevier Essentials). Available from: URL: http://www.blickinsbuch.de/item/feb4a7f3fad7dce8ef19246a95090e6d.

[34] von Elm E, Schreiber G, Haupt CC (2019). Methodische Anleitung für scoping reviews (JBI-Methodologie). Z Evid Fortbild Qual Gesundhwes.

