# Supplementary material for: Transition of patients with recently diagnosed Dementia from inpatient to outpatient setting– a scoping review
Source: BMC Geriatr. 2024 Jan 8;24:37. doi: 10.1186/s12877-023-04638-y (PMC10775549; doi:10.1186/s12877-023-04638-y)
Supplement: Supplementary file 1 — Supplementary Material 1 [file 12877_2023_4638_MOESM1_ESM.pdf]

Transition of patients with recently diagnosed dementia from inpatient to outpatient setting – a scoping review: Search algorithm in the different databases

The search terms were initially created for Pubmed and adapted to the other databases.

Subject 1 "General practitioner": "primary health care"[MeSH Terms] OR "primary care"[All Fields] OR "physicians, family"[MeSH Terms] OR "general pract*"[All Fields] OR "family"[Affiliation] OR "family pract*"[All Fields] OR "family physician*"[Text Word] OR "family doctor*"[All Fields] OR "family med*"[All Fields] OR "general physician*"[All Fields] OR "general doctor*"[All Fields] OR "general med*"[All Fields] OR "primary care physician*"[All Fields] OR "primary care doctor*"[All Fields] OR "primary care med*"[All Fields]

Subject 2 "Dementia": (dementia[tiab] OR Alzheim*[tiab] OR cogniti*[tiab] OR cognitive disorders[all fields] OR memory[tiab] OR memory disorders[all fields] OR dement*[tiab] OR cognitive decline[tiab])

Subject 3 "Diagnosis": "diagnos*"[All Fields]

Subject 4 "Qualitative studies": qualitative research [MeSH] OR qualitative[tiab] OR themes[tiab] OR interview[tiab] OR focus group[tiab] OR observation[tiab] OR videotapes[tiab] OR videotape recording[tiab]

**Pubmed**	((((((((((((((((((((("primary health care"[MeSH Terms]) OR ("primary care")) OR ("physicians, family"[MeSH Terms])) OR ("physicians, primary care"[MeSH Terms])) OR ("primary"[Title/Abstract])) OR ("general pract*")) OR ("family"[Affiliation])) OR ("family pract*")) OR ("family physician*"[Text Word])) OR ("family doctor*")) OR ("family med*")) OR ("general physician*")) OR ("general doctor*")) OR ("general med*")) OR ("primary care physician*")) OR ("primary care doctor*")) OR ("primary care med*")) OR ("physician*"[Title/Abstract])) OR ("practice*"[Title/Abstract])) OR ("gp"[Title/Abstract])) OR ("gps"[Title/Abstract])) AND ((((((((((dementia[Title/Abstract]) OR (alzheim*[Title/Abstract])) OR (cogniti*[Title/Abstract])) OR (cognitive func*)) OR (cognitive impair*)) OR (cognitive disorder*)) OR ("memory"[Title/Abstract])) OR ("memory disorder*")) OR (dement*[Title/Abstract])) OR ("cognitive decline"[Title/Abstract]))) AND ((((diagnos*) OR ("examination*")) OR ("differential diagnos*")) OR ("diagnoses, differential"))) AND ((((((((((("qualitative research"[MeSH Terms]) OR ("qualitative"[Title/Abstract])) OR ("themes"[Title/Abstract])) OR ("interview"[Title/Abstract])) OR ("focus group"[Title/Abstract])) OR ("focus group discussion*"[Title/Abstract])) OR ("observation"[Title/Abstract])) OR ("videotape*"[Title/Abstract])) OR ("videotape recording"[Title/Abstract])) OR ("video recording"[Title/Abstract])) OR ("field observation"[Title/Abstract]))
**Scopus**	(TITLE-ABS-KEY ("primary health care") OR ALL ("primary care") OR TITLE-ABS-KEY ("physicians, family") OR TITLE-ABS-KEY ("physicians, primary care") OR TITLE-ABS-KEY ("primary") OR TITLE-ABS-KEY (general AND pract*) OR TITLE-ABS-KEY (family AND pract*) OR TITLE-ABS-KEY (family AND physician*) OR TITLE-ABS-KEY (family AND doctor*) OR TITLE-ABS-KEY (family AND med*) OR TITLE-ABS-KEY (general AND physician*) OR TITLE-ABS-KEY (general AND doctor*) OR TITLE-ABS-KEY (general AND med*) OR TITLE-ABS-KEY (primary AND care AND physician*) OR TITLE-ABS-KEY (primary AND care AND doctor*) OR TITLE-ABS-KEY (primary AND care AND med*) OR TITLE-ABS-KEY (physician*) OR TITLE-ABS-KEY (practice*) OR TITLE-ABS-KEY ("gp") OR TITLE-ABS-KEY ("gps")) AND (TITLE-ABS-KEY (dement*) OR TITLE-ABS-KEY (alzheim*) OR TITLE-ABS-KEY (cogniti*) OR TITLE-ABS-KEY (cognitive AND func*) OR TITLE-ABS-KEY (cognitive AND impair*) OR TITLE-ABS-KEY (cognitive AND disorder*) OR TITLE-ABS-KEY ("memory") OR TITLE-ABS-KEY (memory AND disorder*) OR TITLE-ABS-KEY ("cognitive decline")) AND (TITLE-ABS-KEY (diagnos*) OR TITLE-ABS-KEY (examination*) OR TITLE-ABS-KEY (differential AND diagnos*)) AND (TITLE-ABS-KEY ("qualitative research") OR TITLE-ABS-KEY ("qualitative") OR TITLE-ABS-KEY ("themes") OR TITLE-ABS-KEY ("interview") OR TITLE-ABS-KEY ("focus group") OR TITLE-ABS-KEY (focus AND group AND discussion*) OR TITLE-ABS-KEY ("observation") OR TITLE-ABS-KEY (videotape*) OR TITLE-ABS-KEY ("videotape recording") OR TITLE-ABS-KEY ("video recording") OR TITLE-ABS-KEY ("field observation")) AND NOT (ALL ("cancer")) AND NOT (ALL (child*)) AND NOT (ALL ("anxiety")) AND NOT (ALL ("end of life")) AND NOT (ALL ("schizophrenia")) AND NOT (ALL ("ADHD")) AND NOT (ALL ("diabetes")) AND (LIMIT-TO (LANGUAGE , "English") OR LIMIT-TO (LANGUAGE , "German"))

**Web of Science**	Query #1 GP<br>(((((((((((((((((((AB=("primary health care")) OR AB=("primary care")) OR ALL=("physicians, family")) OR ALL=("physicians, primary care")) OR AB=("primary")) OR ALL=("general pract*")) OR ALL=("family")) OR ALL=("family pract*")) OR ALL=("family physician*")) OR ALL=("family doctor*")) OR ALL=("family med*")) OR ALL=("general physician*")) OR ALL=("general doctor*")) OR ALL=("general med*")) OR ALL=("primary care physician*")) OR ALL=("primary care doctor*")) OR ALL=("primary care med*")) OR AB=("physician*")) OR AB=("practice*")) OR AB=("gp")) OR AB=("gps")<br>Query #2 Dementia<br>(((((((((AB=("dementia")) OR AB=("alzheim*")) OR AB=("cogniti*")) OR AB=("cognitive func*")) OR AB=("cognitive impair*")) OR AB=("cognitive disorder*")) OR AB=("memory")) OR AB=("memory disorder*")) OR AB=("dement*")) OR AB=("cognitive decline")<br>Query #3 Diagnose<br>(((ALL=("diagnos*")) OR ALL=("examination*")) OR ALL=("differential diagnos*")) OR ALL=("diagnoses, differential")<br>Query #4 Qualitative<br>((((((((((ALL=("qualitative research")) OR AB=("qualitative")) OR AB=("themes")) OR AB=("interview")) OR AB=("focus group")) OR AB=("focus group discussion")) OR AB=("observation")) OR AB=("videotape*")) OR AB=("videotape recording")) OR AB=("video recording")) OR AB=("field observation")
**Cochrane Library**	#1 "primary health care"<br>#2 "primary care"<br>#3 "primary care physician*"<br>#4 "primary care doctor*"<br>#5 "primary care med*"<br>#6 "physicians, family"<br>#7 "physicians, primary care"<br>#8 ("primary"):ti,ab,kw<br>#9 "general pract*"<br>#10 "general physician*"<br>#11 "general doctor*"<br>#12 "general med*"<br>#13 ("physician*"):ti,ab,kw<br>#14 ("practice*"):ti,ab,kw<br>#15 "family"<br>#16 "family pract*"<br>#17 "family physician*"<br>#18 "family doctor*"<br>#19 "family med*"<br>#20 ("gp" OR "gps"):ti,ab,kw<br>#21 {OR #1-#20}<br>#22 ("dementia" OR "dement*"):ti,ab,kw<br>#23 ("alzheim*")<br>#24 ("cog* func*" OR "cog* impair*")<br>#25 ("cogniti*"):ti,ab,kw<br>#26 "cog* disorder*"<br>#27 ("cog* decline"):ti,ab,kw<br>#28 ("memory"):ti,ab,kw<br>#29 "memory disorder*"<br>#30 {OR #22-#29}<br>#31 "diagnos*"<br>#32 "examination"<br>#33 "differential diagnos*"<br>#34 "diagnoses, differential"<br>#35 {OR #31-#34}<br>#36 "qualitative research"<br>#37 ("qualitative"):ti,ab,kw<br>#38 ("themes"):ti,ab,kw<br>#39 ("interview"):ti,ab,kw<br>#40 ("focus group"):ti,ab,kw<br>#41 ("focus group discussion"):ti,ab,kw<br>#42 ("observation"):ti,ab,kw<br>#43 ("videotape*"):ti,ab,kw<br>#44 ("videotape recording"):ti,ab,kw<br>#45 ("video recording"):ti,ab,kw<br>#46 ("field observation"):ti,ab,kw

	#47 {OR #36-#46}<br>#48 #21 AND #30 AND #35 AND #47
**PsychInfo**	SU "primary health care" OR SU "physicians, primary care" OR SU "physicians, family" OR TI "gp" OR AB "gp" OR TI "gps" OR AB "gps" OR TX "primary care" OR TI "primary" OR AB "primary" OR TX "general pract*" OR AF "family" OR TX "family pract*" OR TX "family physician*" OR TX "family doctor*" OR TX "family med*" OR TX "general physician*" OR TX "general doctor*" OR TX "general med*" OR TX "primary care physician*" OR TX "primary care doctor*" OR TX "primary care med*" OR TI "physician*" OR AB "physician*" OR TI "practice*" OR AB "practice*"<br>AND<br>TI ("dementia" OR "dement*" OR "alzheim*" OR "cognitive decline" OR "cogniti*" OR "memory") OR AB ("dementia" OR "dement*" OR "alzheim*" OR "cognitive decline" OR "cogniti*" OR "memory") OR TX "cognitive func*" OR TX "cognitive impair*" OR TX "cognitive disorder*" OR TX "memory disorder*"<br>AND<br>TX "diagnos*" OR TX "examination" OR TX "differential diagnos*" OR TX "diagnoses, differential"<br>AND<br>SU "qualitative research" OR TI (“qualitative" OR "themes" OR "interview" OR "focus group" OR "focus group discussion" OR "observation" OR "videotape*" OR "videotape recording" OR “video recording” OR “field observation”) OR AB (“qualitative" OR "themes" OR "interview" OR "focus group" OR "focus group discussion" OR "observation" OR "videotape*" OR "videotape recording" OR “video recording” OR “field observation”)
**GeroLit**	16. 40 suchen [und] s11 & s10 & s6<br>> 15. 2 suchen [und] s14 & s11 & s10 & s6<br>> 14. 2373 suchen [und] s13 | s12<br>> 13. 10 suchen [und] ([ALL] Alle Wörter) videotap*<br>erweitern ([ALL] Alle Wörter) videotape recording<br>erweitern ([ALL] Alle Wörter) video recording<br>erweitern ([ALL] Alle Wörter) field observation*<br>> 12. 2367 suchen [und] ([ALL] Alle Wörter) qualitative research<br>erweitern ([ALL] Alle Wörter) interview*<br>erweitern ([ALL] Alle Wörter) focus group*<br>erweitern ([ALL] Alle Wörter) observation*<br>> 11. 3530 suchen [und] ([ALL] Alle Wörter) diagnos*<br>erweitern ([ALL] Alle Wörter) differential diagnos*<br>> 10. 14942 suchen [und] s9 | s8 | s7<br>> 9. 918 suchen [und] ([ALL] Alle Wörter) memory<br>erweitern ([ALL] Alle Wörter) memory disorder*<br>> 8. 2335 suchen [und] ([ALL] Alle Wörter) cog* impair*<br>erweitern ([ALL] Alle Wörter) cogniti*<br>erweitern ([ALL] Alle Wörter) cog* disorder*<br>erweitern ([ALL] Alle Wörter) cog* decline<br>> 7. 13038 suchen [und] ([ALL] Alle Wörter) demenz<br>erweitern ([ALL] Alle Wörter) dement*<br>erweitern ([ALL] Alle Wörter) alzheim*<br>erweitern ([ALL] Alle Wörter) cog* func*<br>> 6. 1107 suchen [und] s5 | s4 | s3 | s1<br>> 5. 262 suchen [und] ([ALL] Alle Wörter) family pract*<br>erweitern ([ALL] Alle Wörter) family physician*<br>erweitern ([ALL] Alle Wörter) family doctor*<br>erweitern ([ALL] Alle Wörter) family med*<br>> 4. 161 suchen [und] ([ALL] Alle Wörter) general pract*<br>erweitern ([ALL] Alle Wörter) general physician<br>erweitern ([ALL] Alle Wörter) general doctor<br>erweitern ([ALL] Alle Wörter) general med*<br>> 3. 512 suchen [und] ([ALL] Alle Wörter) primary care med*<br>erweitern ([ALL] Alle Wörter) physician*<br>erweitern ([ALL] Alle Wörter) family physician*<br>erweitern ([ALL] Alle Wörter) familiy doctor*<br>> 2. 342 suchen [und] s1<br>> 1. 342 suchen [und] ([ALL] Alle Wörter) primary health care<br>erweitern ([ALL] Alle Wörter) primary care<br>erweitern ([ALL] Alle Wörter) primary care physician<br>erweitern ([ALL] Alle Wörter) primary care doctor*
**Cinahl**	(TX primary care doctors OR TX primary care physicians OR TX primary care OR TX primary health care OR TX primary healthcare OR TX primary health OR TX family

	doctor OR TX family physician OR TX general medicine OR TX general physician OR TX general practice OR TX general practitioner) AND (TX cognitive function OR TX cognitive functioning OR TX cognitive OR TX alzheimers OR TX alzheimer's OR TX alzheimer's disease OR TX alzheimers disease OR TX alzheimer's disease symptoms OR TX dementia OR TX dementia patients OR TX dementia care OR TX (dementia patients or people with dementia OR TX cognitive decline OR TX cognitive disorders OR TX cognitive impairment OR TX memory loss OR TX memory disorders OR TX memory development OR TX memory decline OR TX memory) AND (diagnos* OR examination* OR differential diagnos* OR diagnosis, differential) AND (qualitative research OR qualitative OR qualitative study OR interview OR focus group OR focus group discussion OR observation OR videotape* OR videotape recording OR field observation OR observational study)
